# Everybody's Page

**Published:** 1889-09-21

**Authors:** 


### Everybody's Page.

Smokeless gunpowder, used for the first time at Spandau manœuvres, where it greatly surprised the military world, was invented by an apothecary in Croatia, named Wallioda Falkenstein.

Saffron, long neglected, is now coming into favour, because ladies have found out that it is the best thing for giving lace curtains and other fabrics the creamy tint that we prefer to the blue-white of our ancestors.

Those women again! All the botany prizes at the Cork School of Science this year, and the chemistry medal, were carried off by a lady. If this is what feminine study of science means, it is time men put a stop to it—if they can.

Occasionally one hears of vaseline not suiting certain skins, making them rough, etc., and when applied to the head taking out the hair. In such cases one may doubt if pure vaseline has been employed. All sorts of impure petroleum compounds are palmed off under that name. A petroleum jelly has been known so impure as to set up inflammation in the wounds to which it was applied.

In 1789 the hospitals of Paris numbered 48, containing accommodation for 20,341 men and women, and 15,000 foundling children. The term hospital, however, then included all that we mean by asylum and almshouse, and charitable work of every description went on under the same roof. As the population of Paris was about 660,000, the proportion of persons assisted by these charities was about one in 182.

Once we saw a simple-minded tobacconist sprinkling his wares with eau de cologne. He said he wanted to perfume the tobacco, not by any means to increase its weight. If he had been wiser he would have put a mixture of musk and powdered tonca beans inside two pieces of blotting-paper, and placed it in a box under the tobacco. After ten days or so of this sweet association, the tobacco would be sufficiently scented.

Twenty tons of arsenic were lately taken to the glass-making districts with as much precaution as if it had been gold; and of course no chemist will sell arsenic without a world of enquiry and proof that it is not wanted for criminal purposes. Yet in Devon and Cornwall anyone who chooses can get almost as much arsenic as he pleases. Within recent years the mineral refuse of the mines there has been found to contain arsenic, and much of this waste is now being worked over to obtain the drug. It would be quite possible for anyone with a turn for poisoning to get as much arsenic as he wanted at the nearest mine.

Mr. G. M'All Theal, in his history of South Africa, gives a rather discouraging account of the thing called civilisation among the Basutos, Bantus, and other native races. On the whole the civilised negro does not seem to be an improvement on the natural man. The simple native is naked, and clean, and never catches cold. He bathes regularly, and then anoints himself with grease and red clay, so that rain runs off him as off a duck. His sophisticated brother is clothed in European garments, which he wears until they drop off, and is a prey to consumption and other diseases. The simple native drinks water, his sophisticated brother swallows vile alcohol whenever he can get it; so that a scoffer might be excused for bidding those who are alarmed by the enormous increase of the Bantus to be of good cheer. "*Continuez, messieurs,*" he might say: "Go on civilising, and you will be introducing more potent checks on increase of population than war and famine ever were."

Dr. A. B. Griffiths, in a communication to the Chemical Society, states that he has extracted salicylic acid from the leaves and stems of tulips, hyacinths, and other plants of the lily order.

The sale of methylated spirit without a license is said to be on the increase in Glasgow, and the excise officers are on the alert. They are less active in the country, where the sale of medicated wines without a license goes on largely.

A lady, probably enlightened by the Maybrick case as to the cosmetic value of arsenic, recently purchased some to "smooth her skin," as she expressed it. Her ideas as to the proper method of its application were vague; she took enough to poison two people, and it was only the timely application of antidotes and emetics that saved her life.

A dangerous habit is making its appearance in Erin, namely, cocaine taking. It is said that a doctor in Dublin has become a positive slave to the habit, consuming from 50 to 80 grains per diem. It appears that he had an attack of pyæmia, or blood-poisoning, some years ago, which has left him most liable to fearful convulsive pains, which he has found the powerful Peruvian alkaloid alleviates.

The result of the prohibition law in the American States where it prevails, is said to be that people who used to go to the public-house for beer or whisky, seek the chemist's instead and ask for—say—tonic bitters. The magistrates here incline to think that something of the same kind is intended when a chemist asks for a spirit license, and catechises the application in much the same way as if he meant to open a tavern.

At the Congress of Dentists in Paris, Dr. E. Rosenthal in an article "Must we Destroy Exposed Pulps?" advocated doing so except in the case of young persons, stating that discolouration of the teeth might be avoided by filling soon after. The nerve killer that he found best was composed of equal parts of iodoform and arsenious acid made into a stiff paste with carbolic acid; this was applied immediately upon the exposure of the nerve pulp.

A good many patients and their friends are apt to look upon the law that forbids the introduction of presents of food or liquor as an unreasonable and cruel restriction. It is well, therefore, that some publicity should be given to the case of Thomas Banbrough, which shows in a very marked manner the necessity for such a rule. Banbrough was recovering from typhoid fever at the London Hospital, when his father visited him, and, contrary to the regulations of the hospital, gave him some grapes. He ate these, with the result that peritonitis supervened, from which he died. Moral: Doctors, nurses, and hospital authorities usually have a reason for what they do.

Conduct is fate, but conduct is the result of character, and character is largely formed by the influences of climate. Man is, if not the creature of his environment, largely affected by it, and the climate which enervates him, makes him languid and dreamy, and leaves him no wants but a hunger, which nature, in the same climate, easily provides the means of satisfying, produces in him special mental characteristic. India, for example, is a land of dreams, the birthplace of the subtlest metaphysic, the home of the transcendental, and at the same time of the utmost sensuality in daily life. The sense of the practical, the balance of mind and body which comes of fighting with hostile elements and conquering them, is hardly ever to be found in the man of the tropics.

